# Application of the Morphological Uterine Scoring Assessment in Ultrasound for Abnormal Uterine Bleeding

**DOI:** 10.7759/cureus.74709

**Published:** 2024-11-28

**Authors:** Sanjay M Khaladkar, Aryaman Dhande, Rohan N Shah, Prajakta P KirdatPatil

**Affiliations:** 1 Radiodiagnosis, Dr. D. Y. Patil Medical College, Hospital and Research Centre, Pune, IND

**Keywords:** abnormal uterine bleeding, adenomyosis, aub, morphological uterus sonographic assessment, musa, ultrasonography, uterine fibroids

## Abstract

Abnormal uterine bleeding (AUB) is a common gynecological condition that disrupts women's health due to irregularities in menstrual frequency, duration, and volume, often resulting in a significant impact on daily life and productivity. Accurate diagnosis of AUB is critical but complicated by its varied etiologies and presentations. Recent advancements in imaging techniques, particularly the Morphological Uterus Sonographic Assessment (MUSA), have enhanced the diagnostic precision of uterine pathologies such as fibroids and adenomyosis. MUSA combines gray-scale sonography, color Doppler, and three-dimensional ultrasound to evaluate uterine abnormalities with standardized terminology, ensuring diagnostic consistency.

This study aimed to assess the efficacy of MUSA in diagnosing and managing AUB. A descriptive observational study was conducted on 50 patients at Dr. D. Y. Patil Medical College and Hospital, focusing on pre- and post-menopausal women with clinically symptomatic AUB. Patients underwent detailed ultrasonography, including both transabdominal and transvaginal scans, to evaluate uterine structures and correlate findings with histopathology.

Results showed that 62% of patients had adenomyosis, while 38% had fibroids. MUSA effectively differentiated between the two conditions based on key ultrasound characteristics such as serosal contour, junctional zone, myometrial wall symmetry, and echogenicity. Adenomyosis cases showed significantly higher rates of heterogeneous echogenicity and asymmetrical myometrial walls compared to fibroids. Statistical analyses revealed high diagnostic sensitivity and specificity for both conditions, with an overall accuracy of 88.9% for adenomyosis and 94.1% for fibroids.

The findings confirm the utility of MUSA in improving diagnostic accuracy and informing management strategies for AUB, particularly in complex cases. The study highlights MUSA as an indispensable tool for clinicians, facilitating enhanced patient outcomes through precise evaluation and treatment of uterine pathologies.

## Introduction

Abnormal uterine bleeding (AUB) encompasses a range of menstrual irregularities that significantly impact women's health, characterized by deviations in the frequency, duration, regularity, and volume of menstrual flow [1]. This condition is prevalent and can profoundly affect daily life, leading to decreased productivity and, in severe cases, necessitating surgical interventions such as hysterectomy. Effective management of AUB hinges on accurate diagnosis, which is often complicated by the diverse etiologies and presentations associated with this condition [2].

In recent years, advancements in imaging technology have greatly enhanced the ability to diagnose and manage AUB. Among these advancements, the Morphological Uterus Sonographic Assessment (MUSA) technique has emerged as a valuable tool for the evaluation of myometrial pathologies [3]. MUSA integrates various ultrasound modalities, including gray-scale sonography, color Doppler imaging, and three-dimensional (3D) ultrasound, to provide a comprehensive assessment of uterine abnormalities. Gray-scale sonography offers detailed images of the uterine structure, enabling the identification of lesions such as fibroids and adenomyosis. Color Doppler imaging further enhances diagnostic accuracy by evaluating blood flow within the uterus, which helps differentiate between different types of myometrial pathologies. Meanwhile, 3D ultrasound provides a more complete view of the uterine anatomy, improving the evaluation of complex lesions and their spatial relationships.

The MUSA technique also incorporates standardized terminology for describing myometrial lesions, ensuring consistency and clarity in reporting. This structured approach facilitates more accurate diagnoses and informs treatment decisions, particularly in cases where traditional imaging methods may be insufficient. By utilizing MUSA, clinicians can achieve a more precise evaluation of uterine pathologies, leading to improved management strategies for AUB [4]. This study aims to assess the utility of MUSA in diagnosing and managing AUB, highlighting its role in enhancing diagnostic accuracy and patient outcomes.

## Materials and methods

An observational descriptive study was conducted in the Department of Radiology, Dr. D. Y. Patil Medical College and Hospital and Research Centre, Pune, from August 2022 to July 2024. A total of 50 participants presenting with AUB with suspected endometrial pathologies were screened using ultrasonography.

All women in pre- and post-menopausal age groups who presented with clinically symptomatic AUB were included in the study. The study excluded women with post-hysterectomy status. Additionally, causes of AUB related to general factors (such as drugs, hormones, and systemic diseases), local factors (including vulval, vaginal, and cervical lesions), and obstetric causes were excluded to focus specifically on endometrial pathologies.

Data collection included demographic details such as age and clinical history related to AUB. USG examinations were performed using the Samsung HS70A (Samsung Electronics Pvt. Ltd., Seoul, South Korea) with PRIME technology. Uterine corpus dimensions (cranio-caudal, antero-posterior, transverse), volume, serosal contour, junctional zone (JZ) type and location, myometrial wall pattern, echogenicity, presence of adenomyosis and fibroids, number, location, volume, rim, shadowing, shape, presence of cysts, hyperechogenic islands, subendometrial lines and buds, myometrial vascularity pattern, USG diagnosis, and histopathological correlation were assessed.

After explaining the procedure to the patient, both transabdominal ultrasonography and transvaginal (TVS) ultrasonography were conducted. TVS offered a detailed assessment of the myometrium but within a limited depth, while transabdominal ultrasonography provided a broader view of the entire pelvic region. The initial TVS involved a dynamic 2D scan of the uterus in two perpendicular planes to evaluate uterine mobility and pinpoint areas of specific tenderness. Sonographic cross-sectional images were used to visualize the arcuate venous and arterial vessels near the outer border of the myometrium.

**Figure 1:**
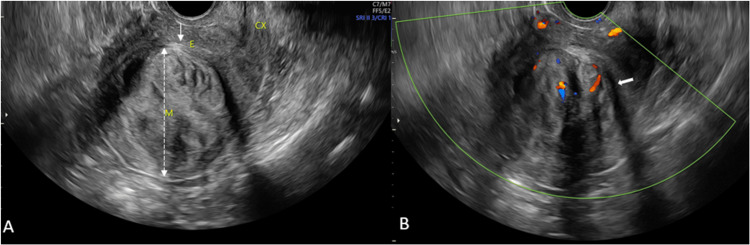
TVS ultrasonography B-mode (A) and color Doppler (B) longitudinal images of a 38-year-old female with complaints of abnormal uterine bleeding and dysmenorrhea A large uterine fibroid (M) is seen along the posterior myometrial wall, intramural in location, with endometrial contact (FIGO 3). It shows heterogeneous echotexture (long dashed double-headed arrow), uniform vascularity, and fan-shaped shadowing (solid arrow), displacing the endometrium (E) anteriorly (line arrow). CX, cervix; FIGO, International Federation of Gynecology and Obstetrics; TVS, transvaginal

As per MUSA [3], fibroids were characterized by a lobulated or regular uterine contour, well-defined lesions, asymmetrical uterine walls, and smooth, round, or oval shapes, often with edge shadows and uniform echogenicity. They exhibited circumferential blood flow and a regular or non-thickened JZ. Fibroids were localized according to the International Federation of Gynecology and Obstetrics (FIGO) classification [2]. In contrast, adenomyosis led to a globally enlarged uterus with ill-defined lesions, irregular or ill-defined contours, mixed echogenicity, fan-shaped shadowing, translesional blood flow, and a thickened, irregular JZ. Adenomyosis also presented with subendometrial lines, buds, and cysts (Figure 1).

In the statistical analysis, descriptive statistics, including mean, standard deviation, range, frequencies, and percentages, were used to summarize demographic and clinical data. Chi-square tests with likelihood ratios were applied to assess significance, with a p-value of <0.05 considered statistically significant.

## Results

Lobulated serosal contours were observed in adenomyosis cases, while regular contours were seen in fibroid cases. Interrupted JZs were found in 13 (40.6%) adenomyosis cases and six (17.6%) fibroid cases, with irregular patterns in 18 (56.3%) adenomyosis cases and none in fibroids. JZs were regular or not assessable in 13 (68.4%) fibroid cases and 10 (31.3%) anterior, seven (21.9%) fundus, 13 (40.6%) global, and three (9.4%) posterior locations in adenomyosis. Asymmetrical myometrial walls were present in 25 (80.6%) adenomyosis cases and four (23.5%) fibroid cases, while symmetrical walls were found in six (19.4%) adenomyosis cases and 15 (76.5%) fibroid cases. Heterogeneous echogenicity was noted in 27 (84.4%) adenomyosis cases and four (23.5%) fibroid cases, and homogeneous echogenicity was seen in four (12.5%) adenomyosis cases and 15 (76.5%) fibroid cases. All differences were statistically significant, with p-values less than 0.001 (Table 1). 

**Table 1:** Comparison of ultrasound characteristics between adenomyosis and fibroid: serosal contour, junctional zone type and location, myometrial wall, and echogenicity LR, likelihood ratio; χ^2^, chi-square

Characteristic	Category	Adenomyosis	Fibroid	Total	LR/χ^2^	p-value
Serosal contour	Lobulated	31	0	31	60.406	<0.001
	Regular	0	19	19		
Junctional zone type	Interrupted	13	6	19	42.708	<0.001
	Irregular	18	0	18		
	Not assessable	0	2	2		
	Regular	0	11	11		
Junctional zone location	Junctional zone regular/not assessable	0	13	13	51.774	<0.001
	Anterior	10	2	12		
	Fundal	0	2	2		
	Fundus	7	0	7		
	Global	13	0	13		
	Posterior	1	2	3		
Myometrial wall	Asymmetrical	25	4	29	17.173	<0.001
	Symmetrical	6	15	21		
Myometrial echogenicity	Heterogeneous	27	4	31	21.809	<0.001
	Homogeneous	4	15	19		

**Figure 2:**
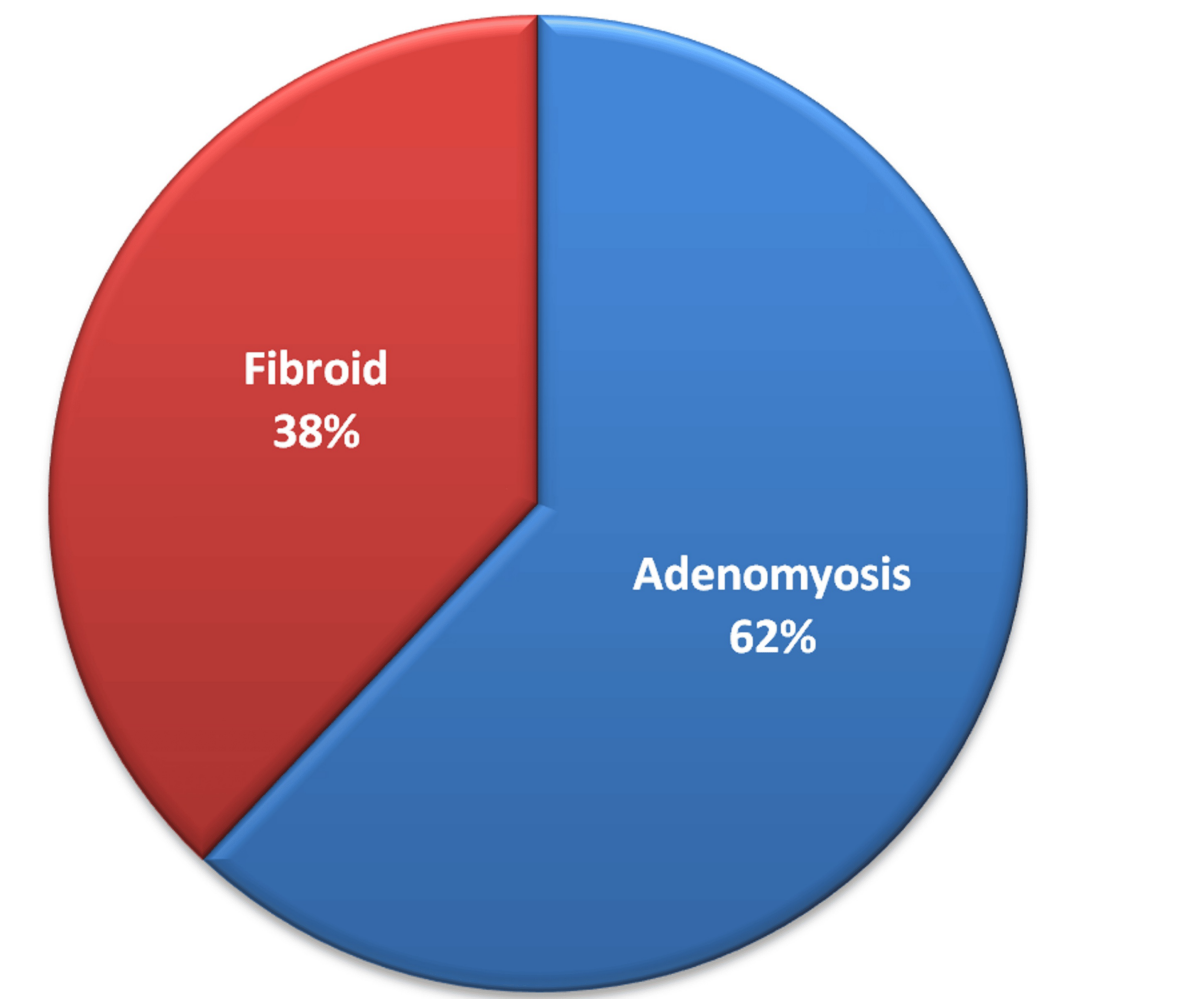
Pie chart showing the distribution of lesions among the study participants

Out of 50 study participants, 31 (62%) had adenomyosis lesions, and 19 (38%) had fibroid lesions (Figure 2). 

**Figure 3:**
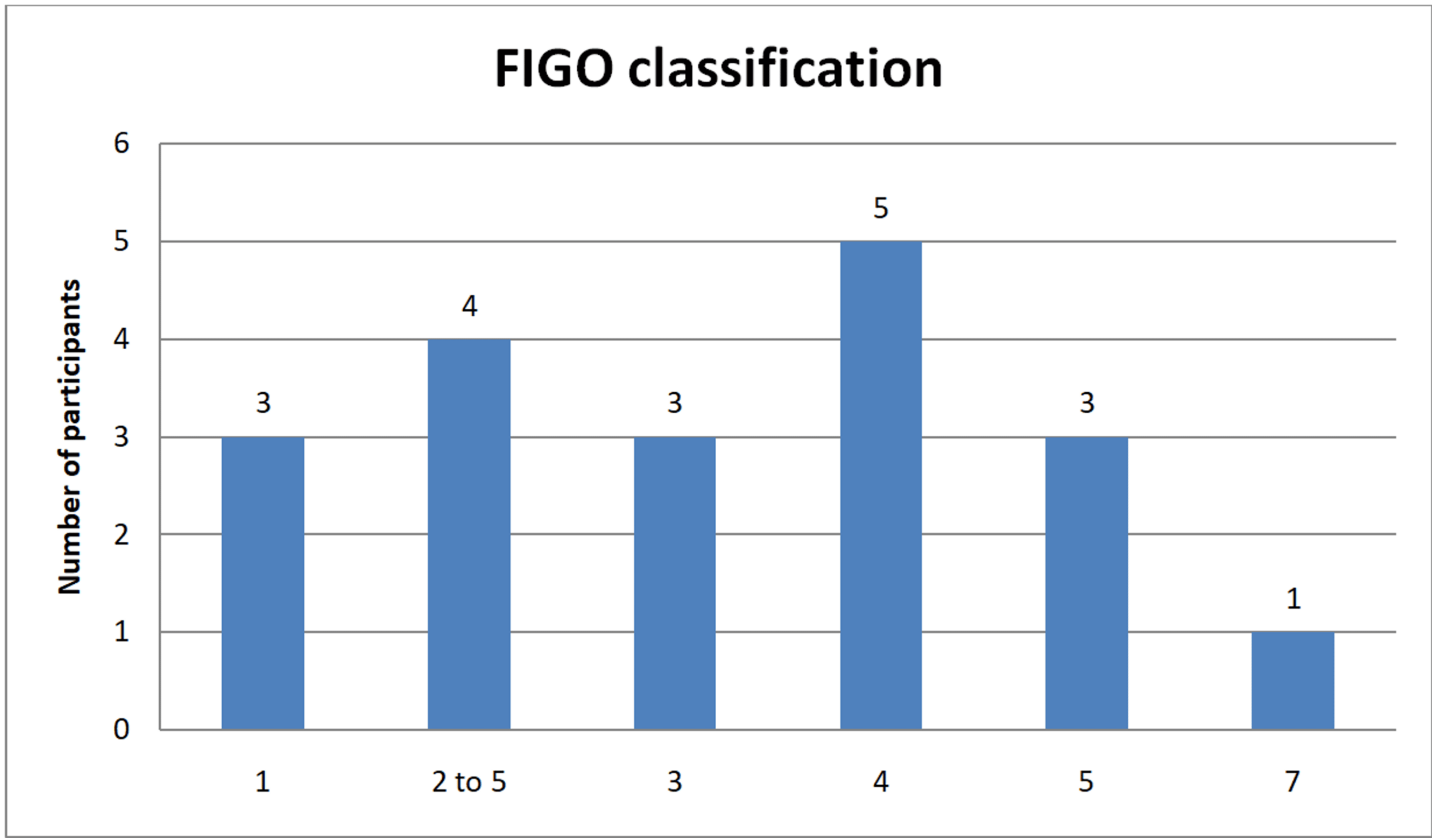
Bar chart showing the distribution of the study participants based on the FIGO classification for fibroids FIGO, International Federation of Gynecology and Obstetrics

Out of the study participants with fibroids, three (15.8%) had submucosal fibroids with intramural extension <50%, four (21.1%) had submucosal, subserosal, and ≥50% intramural fibroids, three (15.8%) had 100% intramural fibroids with endometrial contact, five (26.3%) had 100% intramural fibroids, three (15.8%) had subserosal and ≥50% intramural fibroids, and one (5.3%) had subserosal pedunculated fibroids (Figure 3). 

For fibroid location, 18 cases (31.8%) were anterior, eight cases (13.6%) were fundal, 18 cases (31.8%) were global, and six cases (10.9%) were posterior (p < 0.001). The extent of the lesion was diffuse in 24 cases (43.6%) and localized in 26 cases (47.3%) (p = 0.069). Echogenicity was mixed in 33 cases (60.0%), hyperechogenic in 10 cases (18.2%), and hypoechogenic in seven cases (12.7%) (p < 0.001). The rim characteristics showed hyperechogenicity in nine cases (16.4%), hypoechogenicity in six cases (10.9%), and an ill-defined rim in 35 cases (63.6%) (p < 0.001). Shadowing patterns included edge shadowing in 22 cases (40.0%), fan-shaped in nine cases (16.4%), and internal shadowing in 19 cases (34.5%) (p = 0.004). Shape analysis revealed 34 cases (61.8%) as irregular, four cases (7.3%) as lobulated, and 12 cases (21.8%) as round (p = 0.018) (Table 2). 

**Table 2:** Comparison of ultrasound characteristics between adenomyosis and fibroids LR, likelihood ratio; χ^2^, chi-square

Characteristic	Category	Adenomyosis	Fibroid	Total	LR/χ^2^	p-value
Location of fibroid	Anterior	10	8	18	31.091	<0.001
	Fundal	3	5	8		
	Global	18	0	18		
	Posterior	0	6	6		
Extent of lesion	Diffuse	18	6	24	3.311	0.069
	Localized	13	13	26		
Echogencity	Mixed	25	8	33	16.392	<0.001
	Hyperechogenic	6	4	10		
	Hypoechogenic	0	7	7		
Rim	Hyperechogenic	0	9	9	41.530	<0.001
	Hypoechogenic	0	6	6		
	Ill-defined	31	4	35		
Shadowing	Edge	10	12	22	11.082	0.004
	Fan-shaped	9	0	9		
	Internal	12	7	19		
Shape	Irregular	31	3	34	46.113	0.018

Cysts were present in 28 patients with adenomyosis (89.7%) and six with fibroids (20%), totaling 34 patients (66.7%), while absent in three patients with adenomyosis (10.3%) and 13 with fibroids (43.3%), totaling 16 patients (33.3%) (p < 0.001). Hyperechogenic islands were present in 23 patients with adenomyosis (72.4%) and five with fibroids (16.7%), totaling 28 patients (57.6%), while absent in eight patients with adenomyosis (27.6%) and 14 with fibroids (46.7%), totaling 22 patients (42.4%) (p < 0.001). Subendometrial lines and buds were present in 20 patients with adenomyosis (62.1%) and four with fibroids (13.3%), totaling 24 patients (48%), while absent in 11 patients with adenomyosis (37.9%) and 15 with fibroids (50%), totaling 26 patients (52%) (p = 0.003) (Table 3). 

**Table 3:** Comparison of ultrasound parameters: cysts, hyperechogenic islands, subendometrial lines and buds, and vascularity in adenomyosis and fibroids LR, likelihood ratio; χ^2^, chi-square

Characteristic	Category	Adenomyosis	Fibroid	Total	LR/χ^2^	p-value
Cysts	Absent	3	13	16	18.681	<0.001
	Present	28	6	34		
Hyperechogenic islands	Absent	8	14	22	10.959	<0.001
	Present	23	5	28		
Subendometrial lines and buds	Absent	11	15	26	8.916	0.003
	Present	20	4	24		
Vascularity	Non-uniform	19	6	25	4.160	0.079
	Uniform	12	13	25		

It was indicated that 17 (100%) of the adenomyosis cases were diagnosed with diffuse adenomyosis, whereas no fibroid cases were diagnosed with this condition. In contrast, 14 (100%) of the fibroid cases were diagnosed with fibroid, and no adenomyosis cases were identified as such. Additionally, one (5.9%) adenomyosis case and two (10.5%) fibroid cases were diagnosed with focal adenomyosis. For 11 (28.2%) adenomyosis cases and five (31.3%) fibroid cases, the diagnosis was not available. The differences in diagnoses between adenomyosis and fibroid were statistically significant, with a p-value of <0.001 (Table 4).

**Table 4:** Histopathological diagnosis among the study participants LR, likelihood ratio; χ^2^, chi-square

Characteristic	Category	Adenomyosis	Fibroid	Total	LR/χ^2^	p-value
Histopathological diagnosis	Diffuse adenomyosis	17	0	17	46.532	<0.001
	Fibroid	0	14	14		
	Focal adenomyosis	1	2	3		
	Not available	11	5	16		

The ultrasound screening test for adenomyosis demonstrated high validity with a sensitivity of 90% (correctly identifying 90% of patients with adenomyosis) and a specificity of 87.5% (correctly identifying 87.5% of those without the condition). The positive predictive value (PPV) was 90%, meaning 90% of those diagnosed with adenomyosis by the test were confirmed by histopathology, while the negative predictive value (NPV) was 87.5%, indicating 87.5% of those identified as not having adenomyosis were accurately diagnosed. Overall accuracy was 88.9%. In comparison, the test for fibroids showed a perfect sensitivity of 100% (identifying all patients with fibroids) and a specificity of 90% (correctly identifying patients without fibroids). The PPV was 87.5%, and the NPV was 100%, indicating all patients identified as not having fibroids were correctly diagnosed. The overall accuracy of the fibroid test was higher at 94.1%. 

## Discussion

This study provides significant insights into the differential diagnosis of adenomyosis and fibroids using ultrasound, guided by MUSA criteria. The findings reveal that adenomyosis and fibroids exhibit distinct ultrasound characteristics, confirming the role of detailed morphological assessment in improving diagnostic accuracy.

The presence of lobulated serosal contours exclusively in adenomyosis cases, as opposed to regular contours in fibroid cases, aligns with prior research, which underscores the typical lobulated and distorted outer myometrial contour of adenomyosis due to abnormal tissue growth [5]. In contrast, fibroids generally maintain a regular contour, reflecting their localized and well-circumscribed nature [6].

Regarding the JZ, the study found interrupted and irregular patterns predominantly in adenomyosis cases (40.6% and 56.3%, respectively), while such irregularities were absent in fibroid cases. This supports previous studies emphasizing that JZ abnormalities are characteristic of adenomyosis and indicative of the disruption in the endometrial-myometrial interface [7]. Fibroid cases in this study displayed regular or non-assessable JZs in 68.4% of cases, consistent with their localized growth, which typically does not affect the JZ [8].

The asymmetry of the myometrial walls in adenomyosis cases (80.6%) compared to fibroids (23.5%) is consistent with earlier reports. The diffuse infiltration of adenomyotic tissue into the myometrium often results in an irregular thickening of the walls [9]. This contrasts with fibroids, which, even when multiple, tend to retain symmetry due to their discrete, focal nature [10].

Heterogeneous echogenicity was noted in 84.4% of adenomyosis cases, while fibroids showed homogeneous echogenicity in 76.5% of cases. The heterogeneous echogenicity in adenomyosis reflects the diffuse nature of the disease, with varying densities of ectopic endometrial tissue spread throughout the myometrium [11]. On the other hand, fibroids are more homogeneous due to their well-defined fibrous tissue composition [12].

The detection of cysts, hyperechogenic islands, and subendometrial lines was significantly more prevalent in adenomyosis cases. The presence of these features is characteristic of adenomyosis, as reported in various studies [13]. Subendometrial lines and buds, seen in 62.1% of adenomyosis cases, are indicative of the infiltration of endometrial tissue into the myometrium, a hallmark of adenomyosis. The absence of these features in fibroid cases highlights their diagnostic utility in differentiating between the two conditions [14].

The high sensitivity (90%) and specificity (87.5%) for adenomyosis in this study align with earlier findings that emphasize the reliability of MUSA-based ultrasound criteria for adenomyosis [15]. Previous studies reported sensitivity and specificity values ranging from 80% to 90%, further validating the use of MUSA criteria in clinical settings [16]. The test for fibroids showed even higher diagnostic accuracy with a sensitivity of 100% and specificity of 90%, corroborating studies that report the high diagnostic value of ultrasound for fibroid detection [17].

The results of this study closely mirror findings from Exacoustos et al., who also found a higher incidence of irregular and interrupted JZs in adenomyosis compared to fibroids [18]. Similarly, Van den Bosch et al. reported similar diagnostic accuracy for ultrasound in detecting adenomyosis and fibroids, with sensitivity and specificity values in the high 80s to 90s for both conditions [3]. Our findings also support the conclusions of Dueholm, who highlighted the importance of cysts and heterogeneous echogenicity in diagnosing adenomyosis [19].

While our study found a slightly lower specificity for adenomyosis (87.5%) compared to the perfect sensitivity for fibroids (100%), this could be attributed to the diffuse nature of adenomyosis, which presents more diagnostic challenges than the localized growth of fibroids. However, both conditions exhibited high overall diagnostic accuracy, underscoring the effectiveness of ultrasound as a first-line diagnostic tool.

The study also had some limitations. The small sample size of 50 participants reduced the findings' generalizability, and performing the study at a single site added location-specific biases that may not be applicable in other settings with differing imaging capabilities or clinical experience. The omission of certain sorts of AUB situations limited the application to a subset rather than all of them. Additionally, observer heterogeneity in interpreting ultrasound pictures may have influenced consistency. The reliance on specialized ultrasound technology limited its applicability to other situations without equivalent equipment. As a cross-sectional research, it was unable to determine the cause or long-term patient outcomes, and the absence of extensive histopathological correlation may have hampered diagnosis accuracy, particularly in distinguishing complicated uterine diseases.

## Conclusions

This study reaffirms the diagnostic value of ultrasound in distinguishing between adenomyosis and fibroids, particularly when employing MUSA criteria. The distinct morphological characteristics, such as lobulated serosal contours, asymmetrical myometrial walls, and the presence of subendometrial lines in adenomyosis, provide reliable markers for differentiation. These findings align well with previous research, and the high diagnostic accuracy supports the use of ultrasound as a critical tool in the management of AUB. Further research is needed to refine these criteria, particularly for complex cases involving both adenomyosis and fibroids.
